# Suppressor of Cytokine Signaling 3 in Macrophages Prevents Exacerbated Interleukin-6-Dependent Arginase-1 Activity and Early Permissiveness to Experimental Tuberculosis

**DOI:** 10.3389/fimmu.2017.01537

**Published:** 2017-11-10

**Authors:** Erik Schmok, Mahin Abad Dar, Jochen Behrends, Hanna Erdmann, Dominik Rückerl, Tanja Endermann, Lisa Heitmann, Manuela Hessmann, Akihiko Yoshimura, Stefan Rose-John, Jürgen Scheller, Ulrich Emil Schaible, Stefan Ehlers, Roland Lang, Christoph Hölscher

**Affiliations:** ^1^Infection Immunology, Research Center Borstel, Borstel, Germany; ^2^Department of Microbiology and Immunology, Graduate School of Medicine, Keio University, Tokyo, Japan; ^3^Department of Biochemistry, Christian-Albrechts-University, Kiel, Germany; ^4^Cluster of Excellence Inflammation-at-Interfaces (Borstel-Kiel-Lübeck-Plön), Kiel, Germany; ^5^Medical Faculty, Institute of Biochemistry and Molecular Biology II, Heinrich-Heine-University, Düsseldorf, Germany; ^6^Cellular Microbiology, Research Center Borstel, Borstel, Germany; ^7^Microbial Inflammation Research, Research Center Borstel, Borstel, Germany; ^8^Molecular Inflammation Medicine, Christian-Albrechts-University, Kiel, Germany; ^9^Institute of Clinical Microbiology, Immunology and Hygiene, University Hospital Erlangen, Friedrich-Alexander-University Erlangen-Nuremberg, Erlangen, Germany

**Keywords:** *Mycobacterium tuberculosis*, suppressor of cytokine signaling proteins, mice, knockout, macrophages, arginase I

## Abstract

Suppressor of cytokine signaling 3 (SOCS3) is a feedback inhibitor of interleukin (IL)-6 signaling in macrophages. In the absence of this molecule, macrophages become extremely prone to an IL-6-dependent expression of arginase-1 (Arg1) and nitric oxide synthase (NOS)2, the prototype markers for alternative or classical macrophage activation, respectively. Because both enzymes are antipodean macrophage effector molecules in *Mycobacterium tuberculosis* (*Mtb*) infection, we assessed the relevance of SOCS3 for macrophage activation during experimental tuberculosis using macrophage-specific SOCS3-deficient (LysM^cre^SOCS3^loxP/loxP^) mice. Aerosol infection of LysM^cre^SOCS3^loxP/loxP^ mice resulted in remarkably higher bacterial loads in infected lungs and exacerbated pulmonary inflammation. This increased susceptibility to *Mtb* infection was accompanied by enhanced levels of both classical and alternative macrophage activation. However, high Arg1 expression preceded the increased induction of NOS2 and at early time points of infection mycobacteria were mostly found in cells positive for Arg1. This sequential activation of Arg1 and NOS2 expression in LysM^cre^SOCS3^loxP/loxP^ mice appears to favor the initial replication of *Mtb* particularly in Arg1-positive cells. Neutralization of IL-6 in *Mtb*-infected LysM^cre^SOCS3^loxP/loxP^ mice reduced arginase activity and restored control of mycobacterial replication in LysM^cre^SOCS3^loxP/loxP^ mice. Our data reveal an unexpected role of SOCS3 during experimental TB: macrophage SOCS3 restrains early expression of Arg1 and helps limit *Mtb* replication in resident lung macrophages, thereby limiting the growth of mycobacteria. Together, SOCS3 keeps IL-6-dependent divergent macrophage responses such as *Nos2* and *Arg1* expression under control and safeguard protective macrophage effector mechanisms.

## Introduction

Infection with *Mycobacterium tuberculosis* (*Mtb*) remains a major health threat worldwide (1). The family of suppressor of cytokine signaling (SOCS)-proteins includes eight members (2). These molecules are feedback inhibitors of janus kinases (JAK) signaling pathways downstream of cytokine receptors. During cytokine signaling, SOCS proteins are induced *via* signal transducers and activators of transcription (STAT) molecules and in a negative feedback loop regulate and terminate STAT signaling. Importantly, these proteins determine which STAT factors are activated after cytokine receptor ligation and which downstream signaling events are induced. In particular, suppressor of cytokine signaling 3 (SOCS3) is involved in the regulation of gp130-mediated signaling pathways (3–5). gp130 is a common cytokine receptor chain and is responsible in cooperation with individual receptor subunits—for the recognition of many cytokines, the most important of which is interleukin (IL)-6 (6). In the absence of SOCS3 in macrophages, IL-6 is able to mediate both anti- and pro-inflammatory effects (4, 5, 7). These effects are mediated *via* prolonged STAT3 or STAT1 signaling leading to IL-10- or interferon-gamma (IFN-γ)-like responses, respectively. Eventually, this aberrant gp130-mediated signaling in the absence of macrophage SOCS3 lead to an uncontrolled classical macrophages activation (4). Additionally, a knock down of SOCS3 shifts the activation of macrophages toward an alternative phenotype at the same time (8). Consequently, SOCS3 is essential for regulating the specificity of IL-6 signaling in macrophages.

Macrophages are the main host and effector cells in *Mtb* infection. In experimental animal models, control of mycobacterial replication is strictly dependent on the IL-12-instructed development and infiltration of CD4^+^ T helper type 1 (Th1) cells into the lung (9). The secretion of IFN-γ by Th1 cells eventually induces the so-called classically activated macrophages to express effector molecules central to the anti-mycobacterial immune response such as the inducible nitric oxide synthase (NOS)2 and LRG-47, a member of the 47-kilodalton p47 guanosine triphosphatase family (10, 11). On the other hand, a Th2 immune response induces the arginase-1 (Arg1) expressing so-called alternatively activated macrophages, which counteract protective anti-mycobacterial macrophage effector mechanisms (12–14).

Hence, the activation state of macrophages is critical for the control of mycobacterial growth. *Mtb*-infected macrophages express *Socs3* in a MyD88-dependent manner (15) and during experimental tuberculosis (TB), *Socs3* is induced in lungs of infected mice (13). Previously, SOCS3 in macrophages has been implicated in promoting the IL-12-dependent development of Th1 cells in experimental *Toxoplasma gondii* (16) and *Mtb* (15) infection. To further assess the role of macrophage SOCS3 in experimental TB, we analyzed macrophage responses in macrophage-specific SOCS3-deficient (LysM^cre^SOCS3^loxP/loxP^) mice (5) and give evidence that in the absence of macrophage SOCS3, IL-6 promotes susceptibility to *Mtb* infection by the early induction of Arg1 in resident macrophages. Our study implicates that SOCS3 additionally act as an underappreciated critical component to prevent mycobacterial growth in macrophages.

## Results

### Macrophage-Specific SOCS3-Deficient Mice Are Highly Susceptible to *Mtb* Infection

**Figure 1:**
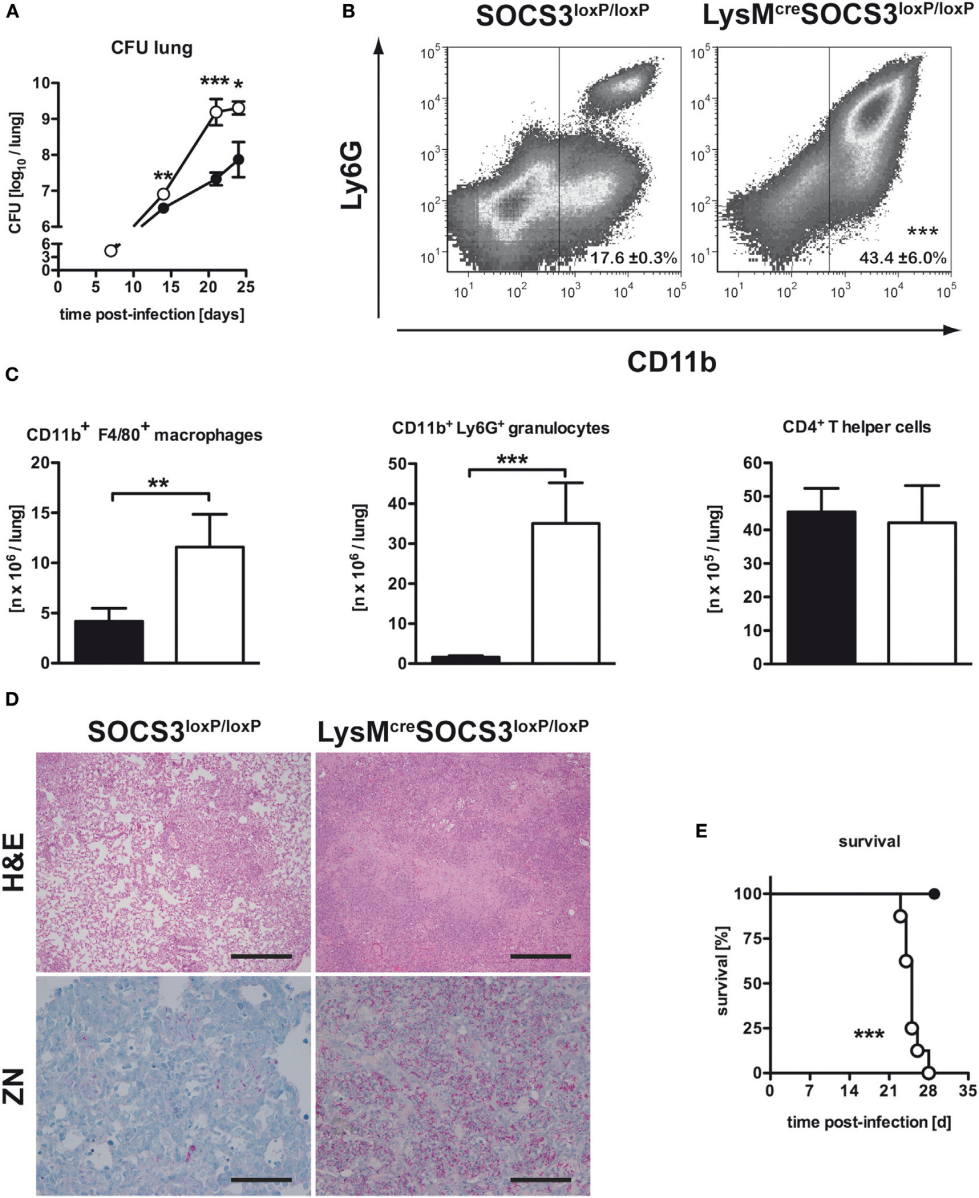
Macrophage-specific suppressor of cytokine signaling 3 (SOCS3)-deficient mice were highly susceptible to *Mtb* infection. Cre-negative SOCS3^loxP/loxP^ control mice (black symbols) and LysM^cre^SOCS3^loxP/loxP^ mice (white symbols) were infected with approximately 1,000 CFU *Mtb via* the aerosol route and lungs were isolated at the indicated time points. **(A)** For mycobacterial colony enumeration assays, aseptically removed lungs were weighed, transferred into PBS containing a proteinase inhibitor cocktail and homogenized. Tenfold serial dilutions of organ homogenates were plated and colonies were counted 3 weeks later. One experiment representative of three performed is shown. **(B,C)** At 23 days of infection, perfused lungs were digested and single-cell suspensions were analyzed by flow cytometry for surface expression of CD11b and Ly6G **(B)**. **(C)** After automated cell counting and flow cytometry of CD11b^+^ F4/80^+^ macrophages, CD11b^+^ Ly6G^+^ granulocytes, and CD4^+^ T cells, absolute numbers of these cell populations were calculated. Data represent means ± SD of at least five mice per group. One experiment representative of two performed is shown. Statistical analysis was performed as described in experimental procedures defining differences between SOCS3^loxP/loxP^ and LysM^cre^SOCS3^loxP/loxP^ mice as significant (***p < 0.001; **p < 0.01). **(D)** Hematoxylin/eosin (H&E, original magnification 100×; scale bar = 1 mm) and Ziehl–Neelsen staining (ZN, original magnification 400×; scale bar = 0.25 mm) of sections from formalin-fixed and paraffin-embedded lungs isolated from mice after 27 days of infection. **(E)** Survival of 10 infected mice per group was monitored. Animals that lost more than 25% of their original body weight were sacrificed. Statistical analysis was performed as described in experimental procedures defining differences between SOCS3^loxP/loxP^ and LysM^cre^SOCS3^loxP/loxP^ mice as significant (***p ≤ 0.001).

To evaluate the effect of macrophage SOCS3 on the outcome of experimental TB, we infected LysM^cre^SOCS3^loxP/loxP^ mice (deficient for SOCS3 in macrophages) and cre-negative littermates. Bacterial loads were determined at different time-points following aerosol *Mtb* infection. Compared to SOCS3^loxP/loxP^ mice, bacterial loads in lungs of LysM^cre^SOCS3^loxP/loxP^ mice were significantly enhanced 21 and 25 days after infection (Figure 1A) confirming the high susceptibility of these mice shown by Carow et al. (15).

### Infiltration of Macrophages and Granulocytes in the Lungs of *Mtb*-Infected LysM^cre^SOCS3^loxP/loxP^ Mice Is Increased

We next assessed the pathological consequences of macrophage-specific SOCS3 deficiency during experimental TB. After 23 days of infection, the total number of cells infiltrating the lungs of *Mtb*-infected LysM^cre^SOCS3^loxP/loxP^ mice was increased threefold when compared to SOCS3^loxP/loxP^ mice (data not shown). The main cell types progressively infiltrating the lungs of macrophage-specific SOCS3-deficient mice were predominantly CD11b^+^ macrophages and granulocytes (Figure 1B). Twenty-three days after *Mtb* infection, their numbers were approximately 3- and 10-fold increased as compared to SOCS3^loxP/loxP^ mice, respectively (Figure 1C). By contrast, the absolute number of CD4^+^ T cells was comparable in the lungs of both SOCS3^loxP/loxP^ and LysM^cre^SOCS3^loxP/loxP^ mice (Figure 1C). Histopathological analysis of lungs taken from LysM^cre^SOCS3^loxP/loxP^ mice confirmed an enhanced inflammatory cell influx (Figure 1D). Organized granuloma formation was absent in cre-positive SOCS3^loxP/loxP^ mice until day 28 (Figure 1D; H&E staining) and this enhanced inflammatory environment alongside the mycobacterial overgrowth (Figure 1D; ZN staining) led to accelerated death of these mice (Figure 1E).

### Inflammatory Immune Responses Are Severely Altered in *Mtb*-Infected LysM^cre^SOCS3^loxP/loxP^ Mice

**Figure 2:**
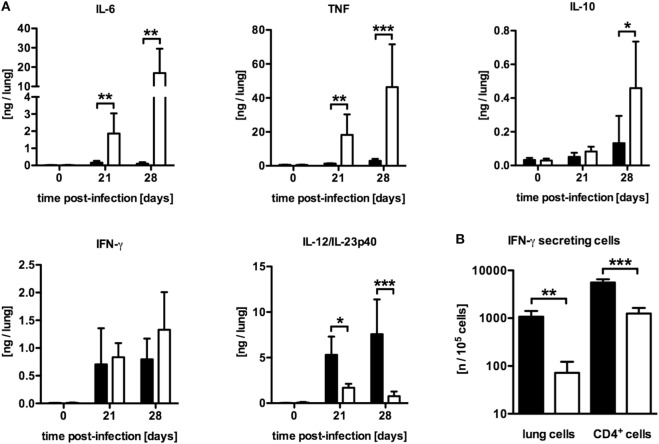
Differentially expressed inflammatory immune responses in *Mtb*-infected LysM^cre^SOCS3^loxP/loxP^ mice. Cre-negative SOCS3^loxP/loxP^ control mice (black symbols) and LysM^cre^SOCS3^loxP/loxP^ mice (white symbols) were infected with approximately 1,000 CFU *Mtb via* the aerosol route and lungs were isolated at the indicated time points. **(A)** Cytokine levels were measured by cytometric bead array (CBA) in lung homogenates and the quantity of cytokines per lung was calculated based on the ratio of lung to sample weight. **(B)** At 23 days of infection, perfused lungs were digested and single-cell suspensions, as well as enriched CD4^+^ T cells, were restimulated with ESAT-6_1–20_. The frequency of IFN-γ-producing cells was determined by an ELISPOT assay. Data represent means ± SD of at least five mice per group. One experiment representative of two performed is shown. Statistical analysis was performed as described in experimental procedures defining differences between SOCS3^loxP/loxP^ and LysM^cre^SOCS3^loxP/loxP^ mice as significant (*p ≤ 0.05; **p ≤ 0.01; ***p ≤ 0.001).

To examine why mycobacteria growth was not controlled in LysM^cre^SOCS3^loxP/loxP^ mice, the induction of pro- and anti-inflammatory cytokines in lung homogenates after *Mtb* infection was characterized by determining the concentrations of IL-6, tumor necrosis factor (TNF), IL-10, IFN-γ, and IL-12/IL-23p40 in a cytometric bead array (CBA) (Figure 2A). Compared to SOCS3^loxP/loxP^ mice, the production of IL-6, TNF, and IL-10 was significantly elevated in the lungs of LysM^cre^SOCS3^loxP/loxP^ mice after *Mtb* infection whereas the amount of IFN-γ was comparable in both cre-negative and cre-positive SOCS3^loxP/loxP^ mice (Figure 2A). In striking contrast, during the course of experimental TB the expression of IL-12/IL-23p40 was significantly reduced in macrophage-specific SOCS3-deficient mice when compared to infected SOCS3^loxP/loxP^ mice (Figure 2A). Because IL-12 is required to promote a protective Th1 immune response (17), we next investigated the frequency of antigen-specific IFN-γ-producing cells using whole cell suspensions or purified CD4^+^ T cells from lungs of infected animals in an ELISPOT assay (Figure 2B). On day 21 of *Mtb* infection, the frequency of IFN-γ-producing CD4^+^ T cells was significantly reduced in the lungs of *Mtb*-infected LysM^cre^SOCS3^loxP/loxP^ mice (Figure 2B). Taken together, these data show a dysregulation of pro- and anti-inflammatory cytokine production in LysM^cre^SOCS3^loxP/loxP^ mice during *Mtb* infection.

Our results are in line with previous studies addressing the impact of a macrophage-specific SOCS3 deficiency on the outcome of experimental toxoplasmosis (16) and TB (15) which implicated the reduced IL-12/IL-23 production and subsequent impaired Th1 immune response to be involved in the impaired control of *Mtb* infection in LysM^cre^SOCS3^loxP/loxP^ mice.

### NOS2 and Arg1 Are Elevated in *Mtb*-Infected LysM^cre^SOCS3^loxP/loxP^ Mice but Arg1 Expression Precedes That of NOS2

**Figure 3:**
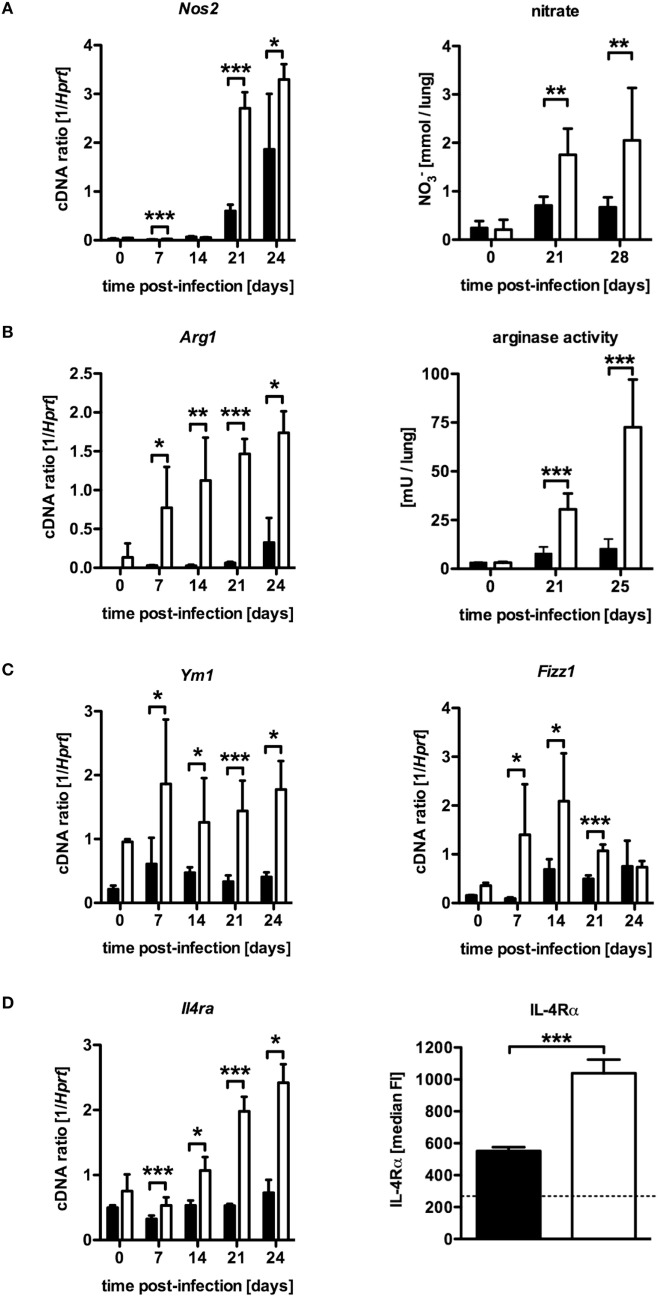
Elevated NOS2 and arginase activity in *Mtb*-infected LysM^cre^SOCS3^loxP/loxP^ mice. Cre-negative SOCS3^loxP/loxP^ control mice (black symbols) and LysM^cre^SOCS3^loxP/loxP^ mice (white symbols) were infected with approximately 1,000 CFU *Mtb via* the aerosol route and lungs were isolated at the indicated time points. **(A)** Gene expression of *Nos2* in lung homogenates was determined by qRT-PCR. To detect RNI, NO_3_ was converted into NO_2_ after deproteination of homogenates. Following the addition of Griess reagents, the content of NO_2_ was determined by photometric measurement. **(B)** Gene expression of *Arg1* in lung homogenates was determined by qRT-PCR. To determine arginase activity, the enzyme was activated and arginine hydrolysis was conducted after the addition of l-arginine. The reaction was stopped and the urea concentration was determined as a degree of arginase activity. **(C)** Gene expression of *Ym1* and *Fizz1* in lung homogenates was determined by qRT-PCR. **(D)** Gene expression of *Il4ra* in lung homogenates and surface expression of the IL-4Rα was determined by qRT-PCR and flow cytometry of CD11b^+^ F4/80^+^ macrophages, respectively. Data represent means ± SD of at least five mice per group. One experiment representative of 2 performed is shown. Statistical analysis was performed as described in experimental procedures defining differences between SOCS3^loxP/loxP^ and LysM^cre^SOCS3^loxP/loxP^ mice as significant (**p ≤ 0.01; ***p ≤ 0.001).

Based on *in vitro* experiments, Carow et al. excluded impaired macrophages effector functions as a cause for the increased susceptibility of *Mtb*-infected LysM^cre^SOCS3^loxP/loxP^ mice (15). The IFN-γ-dependent induction of the effector molecule NOS2 and subsequent production of reactive nitrogen intermediates (RNI) in classically activated macrophages, which are toxic to intracellular *Mtb*, is required for control of mycobacterial growth (10). Hence, the reduced frequency of Th1 cells in LysM^cre^SOCS3^loxP/loxP^ mice might also result in a diminished induction of this enzyme. To evaluate whether a modulated expression of *Nos2* accounts for the increased susceptibility of *Mtb*-infected LysM^cre^SOCS3^loxP/loxP^ mice, we determined the expression of *Nos2* and the production of RNI in lung homogenates of infected mice (Figure 3A). Quantitative real time (qRT)-PCR revealed that 21 and 28 days after *Mtb* infection *Nos2* was considerably induced in lungs of SOCS3^loxP/loxP^ mice (Figure 3A). However, *Nos2* expression was even higher in lung homogenates of infected LysM^cre^SOCS3^loxP/loxP^ mice. To determine the NOS2-dependent production of RNI during experimental TB we quantified the production of nitrate in lung homogenates from SOCS3^loxP/loxP^ and LysM^cre^SOCS3^loxP/loxP^ mice (Figure 3A). In uninfected mice, low amounts of nitrate were detected in lung homogenates. After infection with *Mtb*, the production of nitrates increased in SOCS3^loxP/loxP^ mice. In lung homogenates of infected LysM^cre^SOCS3^loxP/loxP^ mice, nitrate levels were significantly increased by threefold compared to littermate controls. Therefore, it appears that if the increased susceptibility of LysM^cre^SOCS3^loxP/loxP^ mice to experimental TB cannot be attributed to an impaired expression and function of NOS2, other mechanisms may overwrite this normally protective response. Induction of *Nos2* can be counter-regulated by a concomitant Arg1 enzyme activity (12, 13). Arg1, a marker of alternative activation, hydrolyzes l-arginine to urea and l-ornithine and has been discussed to regulate RNI production in macrophages through depletion of l-arginine, the substrate for NOS2 (18, 19). Additionally, Arg1-dependent production of polyamines may promote growth of intracellular pathogens (20, 21). Therefore, we speculated that in LysM^cre^SOCS3^loxP/loxP^ mice, an increased arginase activity in the lungs modulates protective effector mechanisms against *Mtb* usually exerted by classically activated macrophages. A kinetic quantification of gene expression in lung homogenates revealed that *Arg1* is only moderately induced in lungs of SOCS3^loxP/loxP^ mice during the course of *Mtb* infection (Figure 3B). By contrast, *Arg1* expression in LysM^cre^SOCS3^loxP/loxP^ mice was highly induced early during *Mtb* infection. Accordingly, arginase activity in lungs of LysM^cre^SOCS3^loxP/loxP^ mice was also strikingly elevated when compared to enzyme activity in lungs of infected SOCS3^loxP/loxP^ mice (Figure 3B). Importantly, high *Arg1* gene expression precedes the increased gene expression of *Nos2* (Figures 3A,B).

The expression of *Nos2* in infected tissue characterizes classical macrophage activation, which is required for protective macrophage effector responses. By contrast, alternatively activated macrophages are defined by tissue expression of *Arg1* and other markers, such as *Ym1, Fizz1*, and the IL-4Rα-subunit especially during pulmonary inflammation (22). To evaluate whether SOCS3 deficiency generally intensify alternative macrophage activation during *Mtb* infection, gene and surface expression of different markers were quantified by qRT-PCR in lung homogenates and flow cytometry of single-cell suspensions, respectively (Figures 3C,D). Interestingly, LysM^cre^SOCS3^loxP/loxP^ mice constitutively expressed elevated levels of *Ym1* and *Fizz1* in lung homogenates (Figure 3C). Whereas in SOCS3^loxP/loxP^ mice, *Ym1* and *Fizz1* were only marginally stimulated after *Mtb* infection, gene expression of these markers for alternative macrophage activation was highly induced in lung homogenates of *Mtb*-infected LysM^cre^SOCS3^loxP/loxP^ mice. During the course of infection, the expression of these markers was significantly increased in LysM^cre^SOCS3^loxP/loxP^ mice compared to SOCS3^loxP/loxP^ animals. To further evaluate alternative macrophage activation in LysM^cre^SOCS3^loxP/loxP^ mice during experimental TB, we also quantified expression of *Il4ra* by qRT-PCR in lung homogenates and on the single-cell level by flow cytometric analysis of IL-4Rα expression on CD11b^+^ F4/80^+^ lung cells (Figure 3D). Before infection, gene expression was similarly low in both SOCS3^loxP/loxP^ and LysM^cre^SOCS3^loxP/loxP^ mice. After infection with *Mtb*, only LysM^cre^SOCS3^loxP/loxP^ mice showed *Il4ra* gene expression levels that were significantly different from a basal expression in SOCS3^loxP/loxP^ mice. On day 23 of experimental TB, surface expression of the IL-4Rα was significantly increased on macrophages in single lung cell suspensions of LysM^cre^SOCS3^loxP/loxP^ mice when compared to the surface expression on macrophages from infected SOCS3^loxP/loxP^ mice. Our results so far indicate that during *Mtb* infection SOCS3 is involved in regulating early alternative macrophage activation.

**Figure 4:**
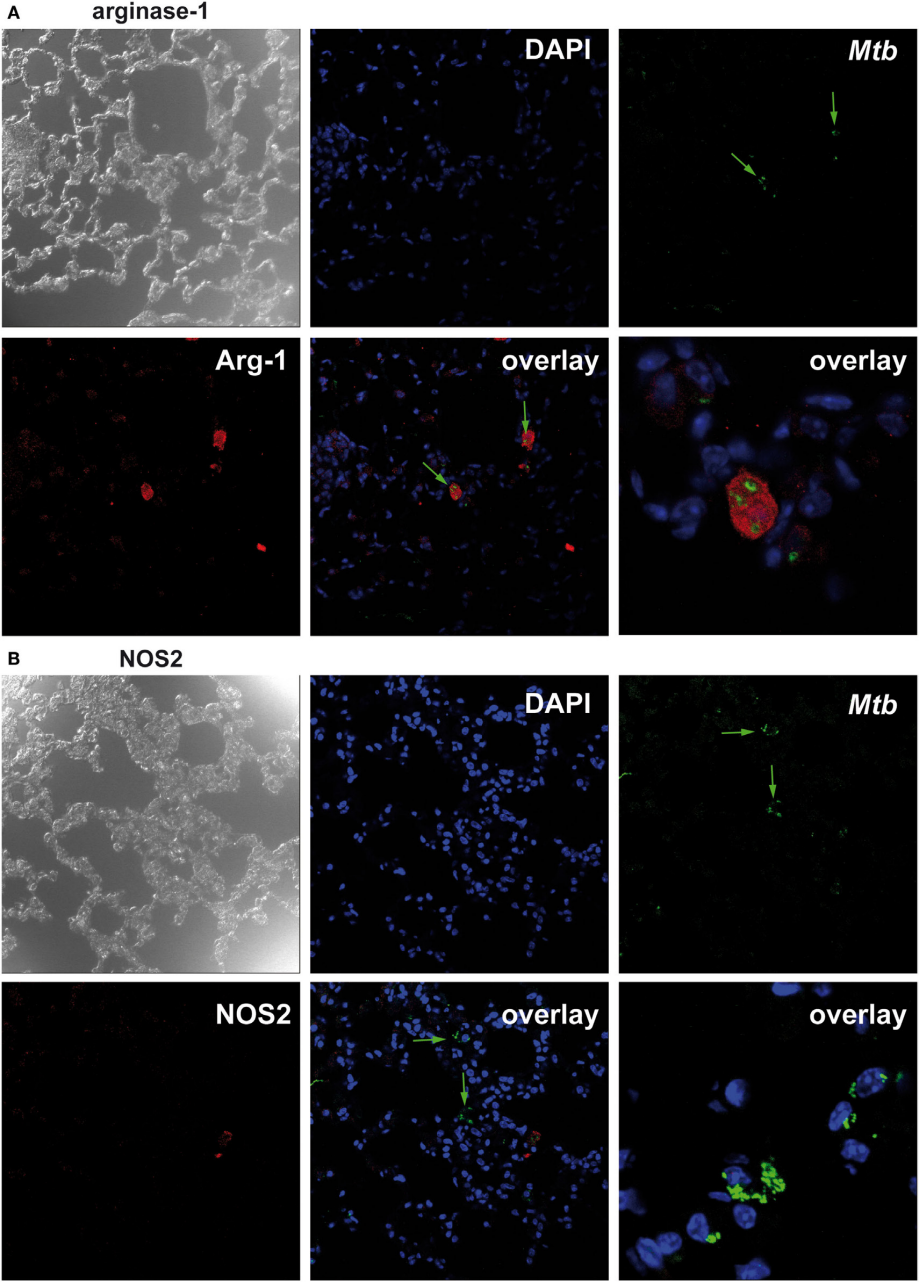
Suppressor of cytokine signaling 3 (SOCS3) deficiency in macrophages favors the initial replication of *Mtb* in arginase-1 (Arg1)-expressing cells. Cre-negative SOCS3^loxP/loxP^ control mice and LysM^cre^SOCS3^loxP/loxP^ mice were infected with approximately 1,000 CFU *Mtb via* the aerosol route and lungs were isolated 1 week later. After antigen retrieval and blocking, deparaffinized formalin-fixed lung sections were incubated with goat anti-Arg1 antibody or rabbit anti-NOS2 antibody. Sections were subsequently incubated with rabbit anti-goat IgG-biotin or goat anti-rabbit IgG-biotin, respectively, followed by an incubation with Streptavidin-Cy5. Finally, sections were co-stained with rabbit anti-*Mtb*-FITC and DAPI. Co-localization of *Mtb* and **(A)** Arg1 or **(B)** NOS2 was determined by confocal microscopy. Data represent representative photomicrographs of at least five mice per group.

Because we found that *Nos2* and *Arg1* are highly expressed in the absence of macrophage SOCS3 but *Arg1* precedes the expression of *Nos2*; we considered that, in LysM^cre^SOCS3^loxP/loxP^ mice, most bacteria might initially and preferentially multiply in *Arg1*-expressing cells. We, therefore, conducted double staining of mycobacteria and NOS2 or Arg1 in lung sections of LysM^cre^SOCS3^loxP/loxP^ mice 1 week after infection with *Mtb*. In SOCS3^loxP/loxP^ mice, NOS2- and Arg1-expressing cells were not detectable at this early time point (data not shown) reflecting the low gene expression of both enzymes (see Figures 3A,B). In lungs of LysM^cre^SOCS3^loxP/loxP^ mice, 1 week after infection *Arg1* precedes *Nos2* gene expression (see Figures 3A,B) and accordingly Arg1-positive cells were abundant (Figure 4A), whereas NOS2-positive cells were hardly detectable (Figure 4B). Importantly, at this early time point, mycobacteria were mostly found in Arg1-expressing cells. Together, these results indicate that in *Mtb*-infected LysM^cre^SOCS3^loxP/loxP^ mice an early exacerbated arginase activity favors the initial replication of *Mtb* particularly in *Arg1*-expressing cells.

### In the Absence of Macrophage SOCS3, *Mtb* Infects Increasingly Arg1-Expressing Resident Macrophages

**Figure 5:**
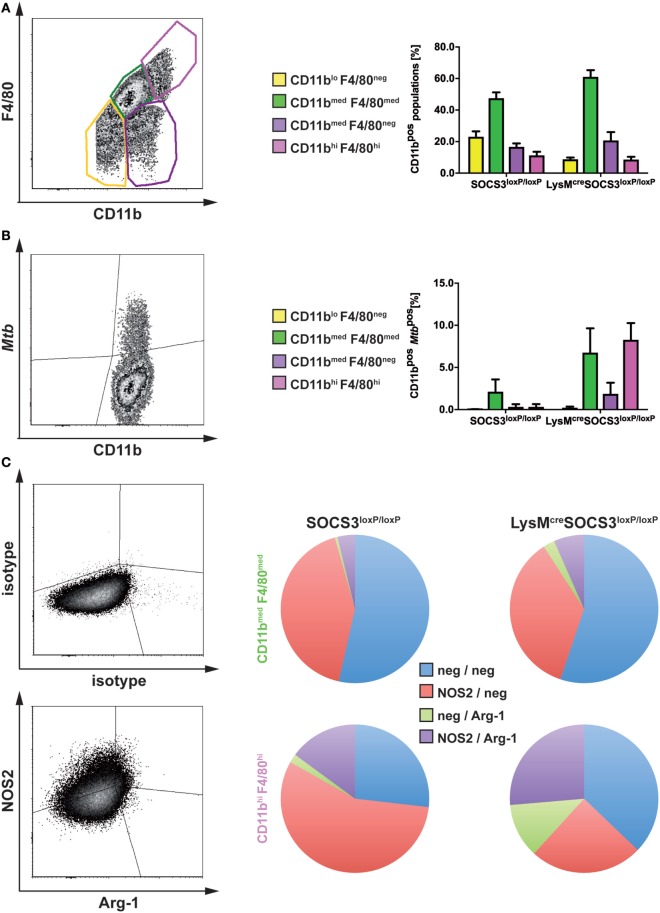
In the absence of macrophage suppressor of cytokine signaling 3 (SOCS3), *Mtb* infects increasingly arginase-1 (Arg1)-expressing resident macrophages. Cre-negative SOCS3^loxP/loxP^ control mice and LysM^cre^SOCS3^loxP/loxP^ mice were infected with approximately 1,000 CFU *Mtb* **(A,C)** or *Mtb*^mCherry^ **(B)** *via* the aerosol route. At 21 or 36 days of infection, respectively, perfused lungs were digested and single-cell suspensions were analyzed by flow cytometry for surface expression of CD11b, F4/80, *Mtb*^mCherry^, and intracellular NOS2 and Arg1. **(A,B)** For flow cytometric analysis of surface markers, single-cell suspensions of lungs were incubated with an anti-FcγRIII/II monoclonal antibody and stained with optimal concentrations of anti-CD11b and anti-F4/80. **(A)** Characterization of different macrophage populations (as defined in the representative plot) in SOCS3^loxP/loxP^ and LysM^cre^SOCS3^loxP/loxP^ mice based on surface expression of CD11b and F4/80. **(B)** The presence of *Mtb* in the macrophage populations defined in **(A)**. **(C)** For intracellular staining of NOS2 and Arg1 in CD11b^med^ F4/80^med^ and CD11b^hi^ F4/80^hi^ cells isolated from lungs of SOCS3^loxP/loxP^ and LysM^cre^SOCS3^loxP/loxP^ mice, single-cell suspensions of lungs were stained for surface markers before permeabilization. Staining of NOS2 and Arg1 was performed using optimal concentrations of anti-NOS2 and anti-Arg1 antibodies. Appropriate isotype controls were used. Data represent means ± SD of three to five mice per group. In **(A and C)**, one experiment representative of two performed is shown.

Having shown that in the absence of macrophage SOCS3 *Mtb* evades in early appearing Arg1-expressing cells, we next analyzed the fate of mycobacteria in different types of macrophages during the course of infection *in vivo*. A defective recruitment of CD11b^med^F4/80^med^ infiltrating macrophages in the lungs of infected mice is associated with susceptibility to *Mtb* (23). The examination of resident CD11b^hi^F4/80^hi^ and infiltrating CD11b^med^F4/80^med^ macrophages in lungs of *Mtb*-infected SOCS3^loxP/loxP^ and LysM^cre^SOCS3^loxP/loxP^ mice (as defined in the representative plot in Figure 5A) revealed a comparable frequency in both strains with a relative proportion of approximately 45–60% infiltrating macrophages and approximately 10% resident macrophages (Figure 5A). Infection of experimental mice with recombinant *Mtb* expressing mCherry enabled us to track bacteria in these cell types by flow cytometry (representative plot in Figure 5B). However, because *Mtb*^mCherry^ was less virulent than wild-type *Mtb*, lungs were isolated on day 36 after infection. Whereas in lungs of SOCS3^loxP/loxP^ mice, *Mtb* was predominantly found in infiltrating macrophages, in LysM^cre^SOCS3^loxP/loxP^ mice mycobacteria were detectable not only in infiltrating CD11b^med^F4/80^med^ but also, to the same extent, in resident CD11b^hi^F4/80^hi^ cells (Figure 5B).

We next determined the relative expression of NOS2 and Arg1 by intracellular staining of both enzymes in resident CD11b^hi^F4/80^hi^ and infiltrating CD11b^med^F4/80^med^ macrophages in lungs of *Mtb*-infected SOCS3^loxP/loxP^ and LysM^cre^SOCS3^loxP/loxP^ mice (Figure 5C). Most of the infiltrating macrophages in lungs of both SOCS3^loxP/loxP^ and LysM^cre^SOCS3^loxP/loxP^ mice were found negative for both, NOS2 and Arg1 or expressed only NOS2. Very few CD11b^med^F4/80^med^ cells expressed Arg1 alone or both, NOS2 and Arg1 (Figure 5C). Approximately 50% of resident CD11b^hi^F4/80^hi^ macrophages in lungs of SOCS3^loxP/loxP^ animals were found single positive for NOS2, about 20% were double positive for NOS2, and Arg-1 and 25% double negative. A small proportion expressed Arg1 only. By contrast, in lungs of LysM^cre^SOCS3^loxP/loxP^ mice the frequencies of Arg1-positive and Arg1/NOS2-positive but also that of Arg1/NOS2-negative CD11b^hi^F4/80^hi^ cells increased at the cost of a decreased relative amount of NOS2-positive cells (Figure 5C).

Together, the absence of SOCS3 is associated with an uncontrolled expression of Arg1 and concomitant enhanced replication of *Mtb* in lung macrophages during experimental TB.

### IL-6 Contributes to Alternative Macrophage Activation and Subsequent Susceptibility and Pathology in *Mtb*-Infected LysM^cre^SOCS3^loxP/loxP^ Mice

**Figure 6:**
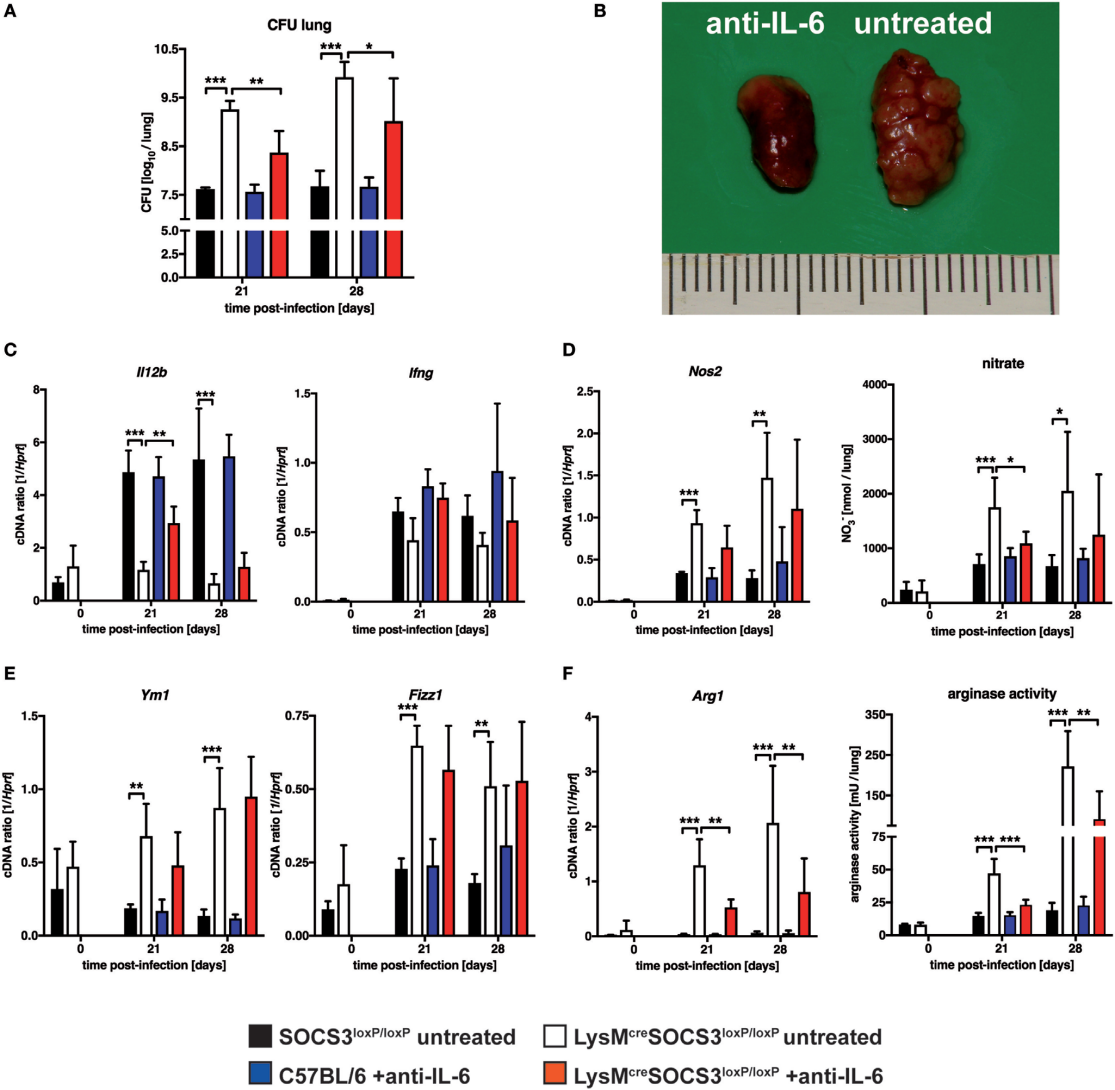
Interleukin (IL)-6-dependent macrophage responses, susceptibility and pathology in *Mtb*-infected LysM^cre^SOCS3^loxP/loxP^ mice. Control (C57BL/6 and Cre-negative SOCS3^loxP/loxP^) mice and LysM^cre^SOCS3^loxP/loxP^ mice were infected with approximately 1,000 CFU *Mtb via* the aerosol route. Two groups, C57BL/6 and LysM^cre^SOCS3^loxP/loxP^ mice, were i.p. injected twice a week with 250 µg of a monoclonal rat anti-mouse IL-6 antibody. Two other groups of cre-negative SOCS3^loxP/loxP^ and LysM^cre^SOCS3^loxP/loxP^ mice were left untreated. Lungs were removed at the indicated time points. **(A)** For mycobacterial colony enumeration assays, aseptically removed lungs were weighed, transferred into PBS containing a proteinase inhibitor cocktail and homogenized. Tenfold serial dilutions of organ homogenates were plated and colonies were counted three weeks later. **(B)** At 28 days of infection, photographs of removed lungs were taken. **(C)** Gene expression of *Il12b* and *Ifng* in lung homogenates was determined by qRT-PCR. **(D)** Gene expression of *Nos2* in lung homogenates was determined by qRT-PCR. To detect RNI, NO_3_ was converted into NO_2_ after deproteination of homogenates. Following the addition of Griess reagents, the content of NO_2_ was determined by photometric measurement. **(E)** Gene expression of *Ym1* and *Fizz1* in lung homogenates was determined by qRT-PCR **(F)** Gene expression of *Arg1* was determined by qRT-PCR. To determine arginase activity, the enzyme was activated and arginine hydrolysis was conducted after the addition of l-arginine. The reaction was stopped and the urea concentration was determined as a degree of arginase activity. Data represent means ± SD of at least five mice per group. Statistical analysis was performed as described in experimental procedures defining differences between control and LysM^cre^SOCS3^loxP/loxP^ mice or untreated and treated animals as significant (*p ≤ 0.05; **p ≤ 0.01; ***p ≤ 0.001).

Suppressor of cytokine signaling 3 is involved in the regulation of gp130-mediated IL-6 signaling and suppresses the induction and maintenance of STAT1- and STAT3-dependent downstream effects (4, 5). We, therefore, determined whether IL-6-dependent signaling was causally involved in the increased susceptibility of LysM^cre^SOCS3^loxP/loxP^ mice during *Mtb* infection *in vivo*. To this end, we neutralized IL-6 by using a monoclonal anti-IL-6 antibody *in vivo*. During experimental TB, IL-6 neutralization had no effect on bacterial loads in lungs of C57LB/6 mice (Figure 6A). In contrast, neutralization of IL-6-dependent signals significantly reduced CFU in lungs of LysM^cre^SOCS3^loxP/loxP^ mice infected for 21 and 28 days with *Mtb*. However, bacterial loads in lungs from anti-IL-6-treated cre-positive SOCS3^loxP/loxP^ mice were still increased compared to infected SOCS3^loxP/loxP^ mice. IL-6 neutralization also greatly ameliorated pathology in LysM^cre^SOCS3^loxP/loxP^ mice infected with *Mtb* for 28 days (Figure 6B). The reduced expression of *Il12b* and *Ifng* in lung homogenates of *Mtb*-infected LysM^cre^SOCS3^loxP/loxP^ mice could only partially be restored by treatment with anti-IL-6 (Figure 6C). Because we show here that macrophage-specific SOCS3 deficiency promotes IL-6-induced classical and alternative macrophage activation during experimental TB *in vivo*, we next assessed whether IL-6 neutralization affected macrophage activation in the lungs of *Mtb*-infected mice. Treatment of C57BL/6 mice with anti-IL-6 had neither an effect on classical macrophage activation (measured by *Nos2* gene expression and nitrate production in lung homogenates; Figure 6D) nor on alternative macrophage activation (determined by *Ym1, Fizz1, Arg1* gene expression, and arginase activity in lung homogenates; Figures 6E,F). IL-6 neutralization reduced classical macrophage activation only to some extent with significantly decreased levels of RNI in lung homogenates of LysM^cre^SOCS3^loxP/loxP^ mice 21 after *Mtb* infection (Figure 6D). In infected SOCS3-deficient mice anti-IL-6 mAb treatment had no effect on gene expression of *Ym1* and *Fizz1* (Figure 6E) but IL-6 neutralization significantly reduced *Arg1* gene expression and arginase activity in lung homogenates of LysM^cre^SOCS3^loxP/loxP^ mice that were infected with *Mtb* for 21 and 28 days (Figure 6F).

Together, these results demonstrate that SOCS3 prevents an IL-6-dependent dysregulated state of macrophage activation *in vivo* that promotes mycobacterial growth.

## Discussion

Reduced expression of SOCS3 has been associated with recurrent and pulmonary disease in TB patients, respectively (24, 25). For the model of experimental TB, Carow et al. convincingly showed that the control of an IL-6-mediated inhibition of IL-12 secretion by SOCS3 in macrophages contributes to the protective effect of SOCS3 (15).

Our results corroborate these findings and additionally show that a lack of macrophage-specific expression of SOCS3 results in a profound susceptibility to *Mtb* infection. The defect in controlling mycobacterial growth might be explained by the impaired IL-12/23p40 production and the decreased frequency of IFN-γ-producing Th1 cells in LysM^cre^SOCS3^loxP/loxP^ mice. This reduced Th1 immune response is in line with reports showing SOCS3 to be responsible for preventing IL-6-dependent suppression of IL-12/23p40 in macrophages and, consequently, for mediating protective Th1 immune responses against *Mtb* (15) or *T. gondii* infection (16).

However, our study is the first to reveal that the increased susceptibility of LysM^cre^SOCS3^loxP/loxP^ mice to *Mtb* infection is associated with strongly elevated levels of both pro- and anti-inflammatory cytokines. Such a dysbalanced inflammatory reaction is likely a direct consequence of the macrophage-specific SOCS3 deletion. SOCS3 binds to the gp130 receptor chain and regulates downstream STAT3-mediated signaling events (26). As a feedback inhibitor of cytokine signaling pathways, it plays a prominent role in limiting and terminating cytokine-induced mechanisms during inflammatory diseases (27–30). When this feedback mechanism is missing, the cytokine response by macrophages is uncontrolled as observed in *Mtb*-infected LysM^cre^SOCS3^loxP/loxP^ mice of the present study. Moreover, the specificity of IL-6 signaling is lost in the absence of macrophage SOCS3 and IL-6 induces aberrant STAT1-dependent pro-inflammatory (IFN-γ-like) and STAT3-mediated anti-inflammatory (IL-10-like) responses (3–5). This regulatory function of SOCS3 is also reflected in *Mtb*-infected LysM^cre^SOCS3^loxP/loxP^ mice of the present study by the concomitantly elevated levels of NOS2 [preferentially induced by IFN-γ/STAT1-mediated signals (31)] and Arg1 as well as the IL-4Rα [both induced to some extent by IL-10/STAT3-dependent signals (32, 33)], respectively.

In light of these findings, macrophage-specific SOCS3 appears necessary to ensure a balanced inflammatory immune response during experimental TB. The absence of SOCS3 in macrophages has detrimental consequences after infection with *Mtb* because excessive cytokine production exacerbated inflammation-induced lung pathology. The present study revealed an elevated infiltration of macrophages and granulocytes into the lungs of *Mtb*-infected LysM^cre^SOCS3^loxP/loxP^ mice resulting in a very rapid loss of functional alveolar space and subsequent early death presumably due to respiratory failure. Hence, macrophage SOCS3 efficiently controls potentially detrimental inflammation-induced pathology early after *Mtb* infection.

Deficiency in macrophage SOCS3 resulted in an utterly immunocompromised phenotype characterized by a strikingly enhanced susceptibility to *Mtb* infection, even in the face of a heightened overall inflammatory response. The exacerbated bacterial loads in LysM^cre^SOCS3^loxP/loxP^ mice might be a consequence of the strikingly increased neutrophil infiltration because susceptibility to *Mtb* infection has previously been attributed to enhanced granulocyte recruitment (34). Hence, we cannot rule out a direct effect of the enhanced granulocyte infiltration on the outcome of experimental TB in these mice. The early defect in controlling mycobacterial growth might also be explained by the impaired IL-12/23p40 production and the decreased frequency of IFN-γ-producing Th1 cells in LysM^cre^SOCS3^loxP/loxP^ mice (15). Yet, after infection with *Mtb* the expression of *Nos2* and the production of RNI were rather elevated in the absence of macrophage SOCS3. Therefore, the inability of LysM^cre^SOCS3^loxP/loxP^ mice to restrict mycobacterial growth appears not to be based on a malfunction of these classically activated macrophages. Th2 cytokines, such as IL-4, IL-10, and IL-13, are potent mediators of alternative macrophage activation that counteract protective functions induced in classically activated macrophages (12, 13, 19, 21). However, in the present study, Th2 cytokines could hardly be detected in both, SOCS3^loxP/loxP^ and LysM^cre^SOCS3^loxP/loxP^ mice (data not shown) but analysis of activation markers such as NOS2 (for classical macrophage activation) and *Fizz1, Ym1, Il4ra*, or *Arg1* (for alternative macrophage activation) revealed that in *Mtb*-infected LysM^cre^SOCS3^loxP/loxP^ mice both phenotypes are significantly enhanced as compared to cre-negative SOCS3^loxP/loxP^ mice. Hence, the increased susceptibility of LysM^cre^SOCS3^loxP/loxP^ mice to *Mtb* infection was associated with an elevated alternative macrophage activation.

We and others have shown that Arg1 in alternatively activated macrophages subverts effector mechanisms against intracellular *Mtb* (12, 13, 19). However, the underlying mechanism induced by Arg1 that is key to a decreased control of mycobacterial growth in macrophages is not fully understood. Classical macrophage activation plays a central role in combating infection with *Mtb* through IFN-γ-induced expression of effector molecules such as the NOS2-dependent production of RNI (35). The production of RNI might be counter-regulated by *Arg1* expression as both *Arg1* and *Nos2* share l-arginine as substrate (18). Hence, substrate depletion by either enzyme might be one regulatory mechanism in macrophages. Though *Mtb* infection induced exacerbated arginase activity in the absence of macrophage SOCS3, RNI production was not reduced. This argues against Arg1-mediated depletion of l-arginine to be a crucial factor for the susceptibility of *Mtb*-infected LysM^cre^SOCS3^loxP/loxP^ mice. After *Mtb* infection, SOCS3-deficient mice expressed increased amounts of both NOS2 and Arg1. It appears that two distinct macrophage populations, classically activated and alternatively activated, are induced by *Mtb* infection in the absence of macrophage SOCS3. Whereas classically activated cells are able to control mycobacterial growth by NOS2 expression and subsequent production of RNI, arginase activity in alternatively activated macrophages may support the replication of *Mtb* in a compartmentalized fashion. Because in *Mtb*-infected LysM^cre^SOCS3^loxP/loxP^ mice an elevated arginase activity precedes the increased induction of NOS2 mycobacteria were mostly found in Arg1-expressing cells at early time points of infection. This differential expression of Arg1 and NOS2 appears to favor the initial replication of *Mtb* particularly in Arg1-expressing cells. In our study, *Mtb* predominantly infected infiltrating macrophages. In LysM^cre^SOCS3^loxP/loxP^ mice, however, mycobacteria were detectable not only in infiltrating but also to a similar extent in resident cells. Moreover, whereas infiltrating macrophages of both SOCS3^loxP/loxP^ and LysM^cre^SOCS3^loxP/loxP^ mice displayed a similar expression pattern of Arg1 and NOS2, SOCS3-deficient resident macrophages consisted of increased proportions of Arg1-expressing cells. Given that alternatively activated macrophages (measured by the gene expression of *Arg1, Ym1*, and *Fizz1*) are already present in the lungs of LysM^cre^SOCS3^loxP/loxP^ mice before infection these mice appear to be constitutively permissive for *Mtb*. As a consequence, the hyperinflammatory phenotype of LysM^cre^SOCS3^loxP/loxP^ mice may have been expedited by this initial susceptibility. Together, SOCS3 restrains an uncontrolled early expression of Arg1 and is necessary for controlling infection of lung macrophages during experimental TB.

Suppressor of cytokine signaling proteins have been shown to have multiple functions in shaping macrophage responses. However, not much is known about the specific impact of SOCS molecules on classical and alternative macrophage activation. Whereas SOCS1 has been described to be crucial for IL-4-induced *Arg1* expression (36), SOCS3 is involved in promoting classical activation (8). Classical macrophage activation is mostly facilitated by the IFN-γ–STAT1 pathway but alternative macrophage activation and *Arg1* expression is mainly induced by the Th2 cytokines IL-4 and IL-13 *via* STAT6- or IL-10 through STAT3-mediated signals (22, 37). Our *in vivo* study confirms previous *in vitro* findings that after IL-6 ligation SOCS3 deficiency results in the preferential induction of STAT1-dependent responses of classical macrophage activation such as the expression of *Nos2* (4). Various markers of alternative macrophage activation including *Arg1* appear to be differentially induced by IL-4/IL-13 and IL-10. IL-4Rα-mediated signals promote full alternative activation. By contrast, IL-10 directly induces *Il4ra* (thereby indirectly enhancing IL-4/IL-13-dependent alternative activation) and *Arg1* but not other markers expressed by alternatively activated macrophages (33). In addition to IL-4, IL-13, and IL-10, IL-6 has recently been shown to specifically induce *Arg1* in macrophages (38). Because SOCS3 regulates signaling at the gp130 receptor chain, the expression of IL-6-induced genes such as *Arg1* is enhanced in SOCS3-deficient macrophages (38). The present *in vivo* study shows for the first time that after infection with *Mtb* the absence of macrophage SOCS3 resulted in exacerbated alternative macrophage activation with strikingly increased levels of *Fizz1, Ym1, Il4ra*, and *Arg1*.

We cannot formally exclude that the reduced IL-12 expression and subsequent impaired Th1 immune response account for the uncontrolled alternative macrophage activation in *Mtb*-infected LysM^cre^SOCS3^loxP/loxP^ mice. It was shown during experimental toxoplasmosis that supplementation with recombinant IL-12 corrects the susceptible phenotype in macrophage-specific SOCS3-deficient animals (16). Along this line, Carow et al. failed to increase the already enhanced bacterial loads in *Mtb*-infected LysM^cre^SOCS3^loxP/loxP^ mice by depleting CD4 T cells and concluded that macrophage SOCS3 does not directly impair innate effector responses (15). In our hands, however, injection of IL-12 did not ameliorate the course of *Mtb* infection in LysM^cre^SOCS3^loxP/loxP^ mice (Figure S1 in Supplementary Material) which may imply that macrophage-specific SOCS3 deficiency does indeed affect effector mechanisms against *Mtb* in macrophages. Although Carow et al. showed that SOCS3-deficient macrophages were in fact able to restrict the growth of *Mtb* in response to IFN-γ, they did not analyze the effect of IL-6 on the expression of *Arg1* and mycobacterial growth in LysM^cre^SOCS3^loxP/loxP^ macrophages (15). However, during *Mtb* infection the key player in the absence of macrophage SOCS3 is IL-6. *In vitro*, SOCS3 has been shown to prevent the development of alternative macrophage activation and the addition of IL-6 to SOCS3-deficient macrophages increases the expression of *Arg1* (8, 38) (data not shown). Hence, by way of an abnormal gp130-dependent signal transduction in SOCS3-deficient macrophages, the presence of IL-6 may still directly affect effector mechanisms of macrophages against *Mtb*. Our hypothesis is supported by experiments of the present study in which depletion of IL-6 in *Mtb*-infected LysM^cre^SOCS3^loxP/loxP^ mice resulted in a significant reduction of bacterial loads and lung pathology. This was accompanied by a reduced expression and activity of Arg1 rather than by a corrected Th1 immune response. Interestingly, IL-6 depletion had no effect on gene expression of *Ym1* and *Fizz1* corroborating previous findings that IL-6 specifically mediates the STAT3-dependent expression of *Arg1* in the first place (38). However, we could exclude that the differential induction of *Ym1* and *Fizz1* is mediated through STAT6 because inhibition of IL-6 also reduces gene expression levels of *Il4ra* (data not shown). Hence, these markers for alternative macrophage activation may be indirectly induced after *Mtb* infection by the hyperinflammatory phenotype in the absence of macrophage SOCS3. Together, our *in vivo* study revealed that SOCS3 is also key in specifically controlling IL-6-mediated *Arg1* expression in macrophages.

We interpret our findings to indicate that in experimental *Mtb* infection SOCS3 does not act in a predetermined, biased way. In addition to promoting protective Th1 immune responses, SOCS3 also keeps IL-6-dependent divergent macrophage responses such as *Nos2* and *Arg1* expression under control. As a consequence, if macrophage SOCS3 is absent preferential replication of mycobacteria in *Arg1*-expressing macrophages and a dysbalanced inflammatory response result in early death. The early appearance of these permissive macrophages is not controlled due to the impaired Th1 immune response. In addition, SOCS3 deficiency allows the immediate exacerbation of Arg1 expression in *Mtb*-infected macrophages. As a consequence, macrophage-specific SOCS3 is essential to eventually safeguard protective macrophage effector mechanisms.

## Materials and Methods

### Ethics Statement

All animal experiments performed were in accordance with the German Animal Protection Law and were approved by the Animal Research Ethics Board of the Ministry of Environment, Kiel, Germany.

### Mice

SOCS3^loxP/loxP^ mice were backcrossed 10 generations to C57BL/6 under specific-pathogen-free conditions at the Technical University of Munich (Germany) and the University of Erlangen (Germany). Mating with LysM^cre^ mice on the C57BL/6 genetic background produced LysM^cre^SOCS3^loxP/loxP^ and cre-negative SOCS3^loxP/loxP^ littermates. In addition, in some experiments, C57BL/6 mice (Charles River, Sulzfeld, Germany) were used as controls.

### Infection with *Mtb*

*Mycobacterium tuberculosis* H37rv or recombinant *Mtb*^mCherry^ (39) were grown in Middlebrook 7H9 broth (Difco, Detroit, MI, USA) supplemented with Middlebrook OADC enrichment medium (Life Technologies, Gaithersburg, MI, USA), 0.002% glycerol, and 0.05% Tween 80. Midlog phase cultures were harvested, aliquoted, and frozen at −80°C. After thawing, viable cell counts were determined by plating serial dilutions of the cultures on Middlebrook 7H10 agar plates followed by incubation at 37°C. Before infection of experimental animals, stock solutions of *Mtb* were diluted in sterile distilled water to a defined concentration and pulmonary infection was performed using an inhalation exposure system (Glas-Col, Terre-Haute, IN, USA).

### Colony Enumeration Assay and Histology

Bacterial loads in lungs were evaluated at 3 and 4 weeks of infection. Lungs were removed aseptically, weighed, and transferred into PBS containing a proteinase inhibitor cocktail (Roche Diagnostics, Mannheim, Germany) and homogenized using the FastPrep™ System (MP Biomedicals, Solon, OH, USA). Tenfold serial dilutions of organ homogenates were plated onto Middlebrook 7H10 (Life Technologies, Darmstadt, Germany) agar plates containing 5% glycerine (Applichem, Darmstadt, Germany) and 10% heat-inactivated bovine serum (Biowest, Nuaillé, France) and incubated at 37°C for 21 days.

For histology, one lung lobe per mouse was fixed in 4% formalin-PBS, set in paraffin blocks, and sectioned (2–3 µm). Histopathological analyses were performed using standard protocols for hematoxylin/eosin staining. Acid-fast bacilli were detected using a modified Ziehl–Neelsen protocol. For the immunohistochemical detection of Arg1 and NOS2 (Upstate, Lake Placid, NY, USA) tissue sections were deparaffinized and pressure cooked in 10 mM citrate buffer, pH 6. After peroxidase quenching with 3% H_2_O_2_/TBS and blocking with 3% BSA, sections were incubated with primary antibodies against Arg1 (Santa Cruz Biotechnology, Santa Cruz, CA, USA) or NOS2 (Upstate) overnight followed by the incubation with HRP-conjugated goat-anti-rabbit secondary antibody (Dianova, Hamburg, Germany). Development was performed by using Elite ABC Kit (Vector, Burlingame, CA, USA) and diaminobenzidine (Vector).

### Quantitative Real Time PCR

Weighed lung samples before and at different time points of infection with *Mtb*, were homogenized in 4 M guanidinium-isothiocyanate buffer and total RNA was extracted by acid phenol extraction. cDNA was obtained using murine moloney leukemia virus (MMLV) reverse transcriptase (Superscript II, Invitrogen, Karlsruhe, Germany) and oligo-dT (12–18mer; Sigma) as a primer. Quantitative PCR was performed on a Light Cycler (Roche). Data were analyzed employing the “2nd Derivate Maximum method” and “Standard Curve method” using hypoxanthine-guanine phosphoribosyl transferase (*Hprt*) as a housekeeping gene to calculate the level of gene expression in relation to *Hprt*. The following primer and probe sets were employed: *Arg1*: sense 5′-CCT GAA GGA ACT GAA AGG AAA-3′, antisense 5′-TTG GCA GAT ATG CAG GGA GT-3′, probe 5′-TTC TTC TG-3′; *Fizz1*: sense 5′-TAT GAA CAGATG GGC CTC CT-3′, antisense 5′-GGC AGT TGC AAG TAT CTC CAC-3′, probe 5′-GGC AGG AG-3′; *Hprt*: sense 5′-TCC TCC TCA GAC CGC TTT T-3′, antisense 5′-CCT GGT TCA TCA TCG CTA ATC-3′, probe 5′-AGT CCA G-3′; *ifng*: sense 5′-atc tgg agg aac tgg caa aa-3′, antisense 5′-ttc aag act tca aag agt ctg agg ta-3′, probe 5′-CAGAGCCA-3′; *Il4ra*: sense 5′-GAG TGG AGT CCT AGC ATC ACG-3′, antisense 5′-CAG TGG AAG GCG CTG TAT C-3′, probe 5′-CTT CCA GC-3′; *Il12b*: sense 5′-atc gtt ttg ctg gtg tct cc-3′, antisense 5′-gga gtc cag tcc acc tct aca-3′, probe 5′-agc tgg ag-3′; *Nos2*: sense 5′-CTT TGC CAC GGA CGA GAC-3′, antisense 5′-TCA TTG TAC TCT GAG GGC TGA C-3′, probe 5′-AGG CAG AG-3′; *Ym1*: sense 5′-GAA CAC TGA GCT AAA AAC TCT CCT G-3′, antisense 5′-GAG ACC ATG GCA CTG AAC G-3′, probe 5′-GGA GGA TG-3′.

### Quantification of Cytokine Production

The concentrations of cytokines in lung homogenates from uninfected and infected mice were determined by a cytometric bead array (CBA) (BD Biosciences, Heidelberg, Germany) as described (40). IL-6 was analyzed using a CBA mouse-flex-set (BD Biosciences). To determine TNF, IL-12/IL-23p40, IL-10, and IFN-γ, beads were conjugated with purified antibodies (BD Bisosiences) using a functional-bead-conjugation buffer set following the manufacturer’s instructions (BD Biosciences). The quantity of cytokines per lung was calculated based on the ratio of lung to sample weight.

### Arginase Activity and Nitrate Production in Lung Homogenates

To determine arginase activity in murine tissue, weighed pieces of organs were homogenized in 100 µl of 0.1% Triton X-100 (Sigma) containing a protease inhibitor cocktail (Roche). 50 µl of 10 mM MnCl_2_ (Merck, Darmstadt, Germany) and 50 mM Tris–HCl (Merck) were added to all samples and the enzyme was activated by heating for 10 min at 55°C. Arginine hydrolysis was conducted by incubating 25 µl of the activated lysate with 25 µl of 0.5 M l-arginine (Merck) at 37°C for 60 min. The reaction was stopped with 400 µl of H_2_SO_4_ (96%)/H_3_PO_4_ (85%)/H_2_O (1/3/7, v/v/v). As a degree of arginase activity, the urea concentration was measured at 540 nm after addition of 25 µl α-isonitrosopropiophenone (Sigma; dissolved in 100% ethanol) followed by heating at 95°C for 45 min. One unit of arginase activity is defined as the amount of enzyme that catalyzes the formation of 1 µmol urea/min. To detect RNI in uninfected and infected mice, lung homogenates were collected at different time points. After deproteination of homogenates using Micron YM-30 centrifugal filters (Millipore, Schwalbach, Germany), NO_3_ was converted into NO_2_ utilizing a commercial nitrate reductase kit (Cayman; Axxora, Lörrach, Germany). After adding Griess reagents, the content of NO_2_ was determined by photometric measurement reading the absorbance at 540 nm on a microplate reader (Sunrise; Tecan, Männedorf, Switzerland) as previously described (40).

### Confocal Microscopy of *Mtb* in Arg1- and NOS2-Expressing Cells

For fluorescence microscopy, deparaffinized formalin-fixed lung sections were boiled for 45 min in 0.01 M citrate buffer at pH 6.0 for antigen retrieval. Endogenous peroxidase activity was blocked by a 10-min incubation in 1% hydrogen peroxide, unspecific antibody binding was blocked by a 30 min incubation in 3% BSA and endogenous biotin or avidin/streptavidin binding proteins were blocked using the Avidin/Biotin blocking kit (Vector Laboratories). Sections were then incubated overnight at 4°C with goat anti-Arg1 antibody (1:50 dilution, Santa Cruz Biotechnologies) or rabbit anti-NOS2 antibody (1:200 dilution, Merck Millipore). Sections were subsequently incubated with rabbit anti-goat IgG-biotin or goat anti-rabbit IgG-biotin (both Dianova), respectively, followed by an incubation with Streptavidin-Cy5 (Dianova). Finally, sections were co-stained with rabbit anti-*Mtb*-FITC (Biozol) and DAPI (Roche). Fluorescent stainings were analyzed using a TCS SP5 confocal microscope and LAS AF software (both from Leica, Wetzlar, Germany). Color images were produced by pasting each of the original grayscale images (shown in Figure S2 in Supplementary Material) into the red, the green, or the blue channels. Cross-reactivity of goat anti-Arg1 against *Mtb* could be excluded as goat anti-Arg1-dependent signals were absent in lung sections of *Mtb*-infected L-Nil-treated *Arg1*^flox/flox^*Tie2cre* mice, which are deficient for *Arg1* in all macrophage populations.

### Preparation of Single-Cell Suspensions from Infected Lungs

For antigen-specific restimulation and flow cytometric analysis, single-cell suspensions of lungs were prepared from *Mtb*-infected mice at different time points. Lungs were perfused through the right ventricle with warm PBS. Once lungs appeared white, they were removed and sectioned. Dissected lung tissue was then incubated in collagenase A (0.7 mg/ml; Roche Diagnostics, Mannheim, Germany) and DNase (30 µg/ml; Sigma) at 37°C for 2 h. Digested lung tissue was gently disrupted by subsequent passage through a 100 µm pore size nylon cell strainer. Suspensions were depleted of remaining erythrocytes using hypotonic red cell lysis buffer, containing NH_4_Cl and NaHCO_3_. Recovered vital lung cells were counted using an automatic cell counter (ViCell^®^; Beckman Coulter, Krefeld, Germany), diluted in RPMI 1640 medium (Sigma) supplemented with 10% FCS (Life Technologies), 0.05 mM β-mercaptoethanol (Sigma), and penicillin and streptomycin (100 U/ml and 100 µg/ml; Life Technologies) and used for further experiments.

### Flow Cytometric Analysis

For flow cytometric analysis of surface markers, single-cell suspensions of lungs were incubated with an anti-FcγRIII/II monoclonal antibody (clone 2.4.G2) and stained with optimal concentrations of the following specific antibodies: anti-CD11b-eFluor450 (Ebioscience, Frankfurt, Germany), anti-CD11b-V500 (clone M1/70; BD Bioscience), anti-F4/80-Alexa-647 (clone BM8; Invitrogen), anti-F4/80-Pacific Blue (clone BM8; Biolegend), anti-Ly6G-PerCp-Cy5.5 (clone1A8; BD Bioscience), and anti-IL-4Rα-PE (clone mIL-4R-M1; BD Biosciences). For intracellular staining of NOS2 and Arg1, single-cell suspensions of lungs were stained for surface markers before permeabilization with Cytofix/Cytoperm^®^ (BD Bioscience). Staining of NOS2 and Arg1 was performed using optimal concentrations of the following specific antibodies: NOS2-FITC (clone 6/iNOS/NOS type II; BD Bioscience), goat-anti mouse Arg1 (clone V-20; Santa Cruz Biotechnology) and polyclonal donkey-anti-goat IgG-Dylight 650 (Abcam, Cambridge, UK). Appropriate isotype controls were used. Fluorescence intensity was measured using a FACSCanto^®^ II flowcytometer (BD Biosciences). Analysis was performed utilizing the FCS Express^®^ program (*De Novo* Software, Los Angeles, CA, USA).

### ESAT6_1-20_-Specific ELISPOT Assays

Detection of antigen-specific IFN-γ-producing cells from infected lungs was conducted using an ELISPOT assay kit (BD Biosciences). To enrich CD4^+^ T cells, single-cell suspensions were incubated with magnetic CD4 microbeads (Miltenyi, Bergisch Gladbach, Germany) and separated from other cells on a MACS separation unit (Miltenyi). Separated CD4^+^ T cells were collected in Iscoves-modified Dulbeccos medium (IMDM; Life Technologies) supplemented with 10% FCS (Life Technologies), 0.05 mM β-mercaptoethanol (Sigma), and penicillin, and streptomycin (100 U/ml and 100 µg/ml; Life Technologies), counted using a cell counter (ViCell^®^; Beckman Coulter), diluted in IMDM and used for further experiments. For measuring the antigen-specific IFN-γ response in lungs from infected mice, single-cell suspensions or purified CD4^+^ T cells from lungs were seeded in wells of anti-mouse IFN-γ-coated and blocked 96-well multitestplates at an initial concentration of 1 × 10^5^ cells/well in IMDM. After doubling dilutions of these cells were made, mitomycin-D (Sigma)-inactivated splenocytes from uninfected control mice were used as APCs at a concentration of 1 × 10^6^ cells/well. Cells were stimulated with the *Mtb* ESAT6_1–20_ (Research Center Borstel, Germany) at a concentration of 10 µg/ml in the presence of 10 U/ml recombinant mouse IL-2 (Peprotech, Hamburg, Germany). After 20 h of incubation in 5% CO_2_ at 37°C, plates were washed, and biotinylated anti-mouse IFN-γ was used to detect the captured cytokine. Spots were visualized using streptavidin-HRP as substrate. Spots were automatically enumerated using an ELISPOT reader (EliSpot 04 XL; AID, Straßberg, Germany). The frequency of responding cells was determined.

### Neutralization of Endogenous IL-6

To neutralize endogenous IL-6 during experimental TB, 250 µg of a monoclonal rat IgG1, kappa anti-IL-6 antibody (clone MP5-20F3, *InVivo* BioTechServives, Henningsdorf, Germany) were injected i.p. at days −1, 2, 6, 9, 13, 16, 20, and 23 after infection with *Mtb*.

### Statistical Analysis

Statistical analysis was performed using GraphPad Prism version 4.03 (GraphPad Software, San Diego, CA, USA). Quantifiable data are expressed as means of individual determinations and SD. After analyzing for Gaussian distribution, unpaired Student’s *t*-test or the Mann–Whitney test was applied defining different error probabilities (*p ≤ 0.05; **p ≤ 0.01; ***p ≤ 0.001). ANOVA was performed using the Bonferroni multiple comparison test different error probabilities (*p ≤ 0.05; **p ≤ 0.01; ***p ≤ 0.001). Statistical survival analysis was performed using the Log-rank test.

## Author Contributions

ES: designed the study, performed experiments, analyzed the results, and drafted figures and manuscript. MD, JB, and HE: performed experiments, analyzed the results, and drafted figures. DR, TE, LH, and MH: performed experiments. AY, JS, SR-J, and US: provided material. SE and RL: designed the study. CH: designed the study, drafted figures and manuscript.

## Conflict of Interest Statement

The authors declare that the research was conducted in the absence of any commercial or financial relationships that could be construed as a potential conflict of interest.
