# Supplementary material for: Suppressor of Cytokine Signaling 3 in Macrophages Prevents Exacerbated Interleukin-6-Dependent Arginase-1 Activity and Early Permissiveness to Experimental Tuberculosis
Source: Front Immunol. 2017 Nov 10;8:1537. doi: 10.3389/fimmu.2017.01537 (PMC5686055; doi:10.3389/fimmu.2017.01537)
Supplement: Supplementary file 1 [file image_1.pdf]

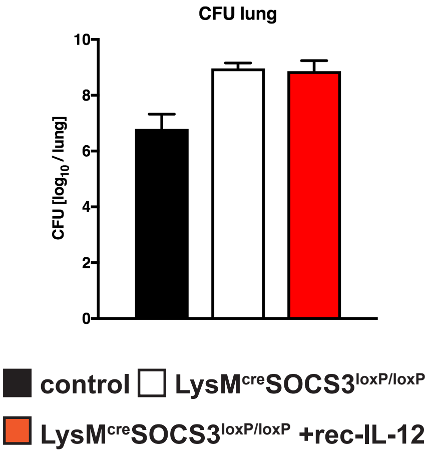

Supplemental figure 1. Injection of recombinant IL-12 has no impact on bacterial replication in Mtb-infected LysM^{cre}SOCS3$^{loxP/loxP}$ mice. Cre-negative SOCS3$^{loxP/loxP}$ control mice and LysM^{cre}SOCS3$^{loxP/loxP}$ mice were infected with approx. 1000 CFU Mtb via the aerosol route. Mice were injected daily with 200 ng of recombinant IL-12 i.p. (Biolegend) or left untreated. After 21 days, mycobacterial colony enumeration assay of removed lungs was performed. Data represent means ±SD of 5 -10 mice.
