# Supplementary material for: Suppressor of Cytokine Signaling 3 in Macrophages Prevents Exacerbated Interleukin-6-Dependent Arginase-1 Activity and Early Permissiveness to Experimental Tuberculosis
Source: Front Immunol. 2017 Nov 10;8:1537. doi: 10.3389/fimmu.2017.01537 (PMC5686055; doi:10.3389/fimmu.2017.01537)
Supplement: Supplementary file 2 [file image_2.pdf]

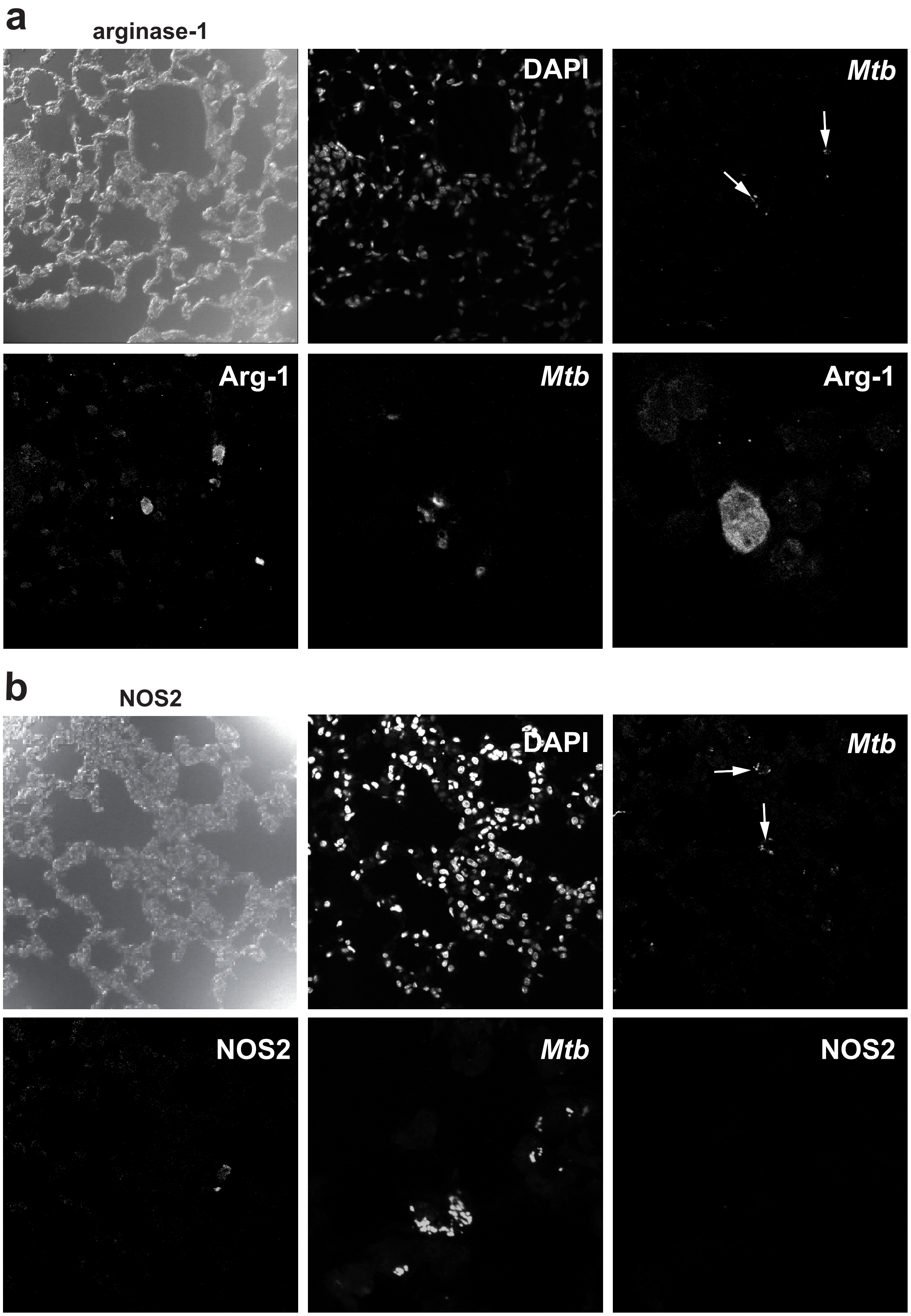

Supplemental figure 2. SOCS3-deficiency in macrophages favors the initial replication of Mtb in Arg1-expressing cells. Cre-negative SOCS3$^{loxP/loxP}$ control mice and LysM^{cre}SOCS3$^{loxP/loxP}$ mice were infected with approx. 1000 CFU Mtb via the aerosol route and lungs were isolated 7 days later. Co-localization of Mtb and (a) Arg1 and (b) NOS2 was determined by confocal microscopy after immunohistochemical staining of formalin-fixed sections. Original grayscale images before pasting into the red, the green, or the blue channels (as shown in supplemental Figure 2). Data represent representative photomicrographs of at least 5 mice per group.
